# Detection of Fatal Potassium Overdose: A Case Report and Review of the Literature

**DOI:** 10.3390/diagnostics13071339

**Published:** 2023-04-04

**Authors:** Gábor Simon

**Affiliations:** Department of Forensic Medicine, Medical School, University of Pécs, 7624 Pécs, Hungary; gabor.simon@aok.pte.hu

**Keywords:** forensic science, forensic pathology, toxicology, potassium overdose, hyperkalemia, post-mortem biochemistry

## Abstract

Potassium overdose usually occurs accidentally, but potassium is also used for judicial executions, assisted death, and, rarely, suicides. In addition to exogenous overdose, various drugs, and renal failure, diabetic ketoacidosis can cause hyperkalemia. Potassium tablets are used in most cases of suicidal potassium overdose. Suicide by intravenous administration of potassium is rare but usually fatal. The author reports a rare case of suicide with potassium infusion. Autopsy and histology findings, along with post-mortem biochemical analysis of different body fluids and fluid from the infusion set, are reported. Previously published reports of potassium overdose were reviewed, and the detection possibilities of potassium overdose are discussed. The detection possibilities of lethal hyperkalemia are very limited since hyperkalemia produces only nonspecific autopsy and histology findings. Post-mortem potassium concentrations are not indicative of ante-mortem potassium concentrations; therefore, post-mortem biochemical analysis has limited value in determining potassium overdose. The best way to prove potassium overdose is via the collection and analysis of circumstantial evidence.

## 1. Introduction

The potential lethality of potassium overdose is well known. The most common cause of potassium overdose is accidental (mixing up drugs or miscalculating the dose [1,2], but it is also used for judicial executions [3,4], assisted death [5], and suicides [6,7,8,9,10,11]. In addition to exogenous overdose, various drugs, renal failure, and diabetic ketoacidosis can also cause hyperkalemia [12,13].

Serum potassium concentration normally varies from 3.8 to 5.5 mEq/L [5]. Hyperkalemia disrupts the depolarization and repolarization of myocardial cells, resulting in diverse types of arrhythmias and cardiac arrest [5,14]. A potassium level of over 8.0 mEq/L will almost always cause diagnostic ECG changes, and at about 9–10 mEq/L potassium, causes ventricular fibrillation and asystole [12]. Hyperkalemia also affects the neuromuscular system, causing symptoms, such as weakness and flaccid paralysis [2,5], and it tends to cause gastrointestinal symptoms, respiratory depression, oliguria, and metabolic acidosis [12]. Apart from the systemic effects of potassium overdose, extravascular leakage of potassium chloride, or direct dermal injection, can lead to local necrosis and chemical burns caused by ischemia due to vascular constriction [15].

The detection of potassium overdose has high forensic relevance, but proving the overdose is difficult. A rare case of potassium overdose by infusion is presented. Only a few such cases of suicide have been reported [6,7], and none have been reported with complete biochemical analysis of different body fluids, toxicology, macroscopic findings, microscopic findings, and analysis of circumstantial evidence. A detailed review of the literature is presented, and the diagnostic possibilities of potassium overdose are discussed.

## 2. Case Report

The body of a 26-year-old male was found in a car located on a forest road. The deceased was sitting in the driver’s seat. An empty infusion bottle hung from the handhold with the labeling of 100 mL, Sodium-Chloride 0.9%. An infusion set had been installed in the bottle, and the other end of the infusion tube was connected to a peripheral venous catheter (PVC) inserted in a peripheral vein on the left forearm of the deceased. The PVC was properly fixed to the skin with an adhesive cannula. Clear fluid was found in the drop chamber of the infusion set. Injection needles, a 24 mL empty syringe, five pieces of empty potassium-chloride ampule (10%, 10 mL), and a suicide note referring to personal issues were found in the car. The victim was a trained paramedic, and his medical history held no severe illnesses. Psychiatric anamnesis was negative.

### 2.1. Autopsy Findings

**Figure 1:**
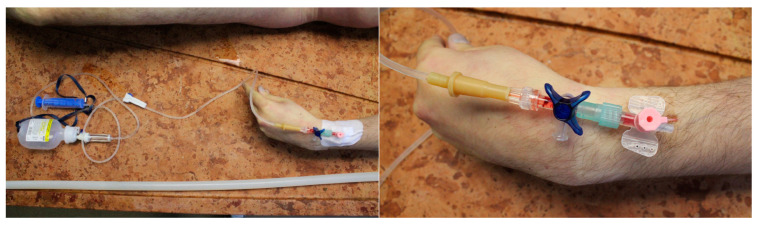
The location of the infusion set. The peripheral venous catheter (PVC) is inserted in a peripheral vein on the left forearm of the deceased. The PVC was properly fixed to the skin with adhesive cannula.

**Figure 2:**
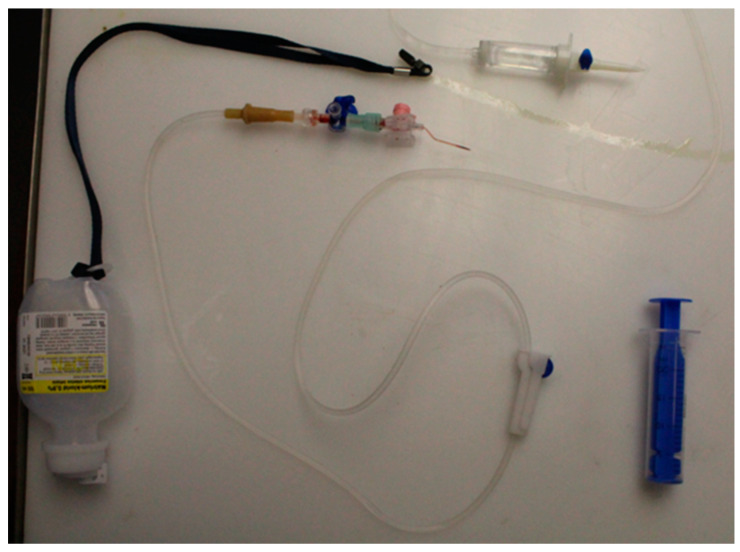
The Infusion set after removal.

A complete medico-legal autopsy was performed 70 h after the body was found. At the beginning of the autopsy, the infusion set was still connected to the deceased (Figure 1 and Figure 2). The body was 181 cm in length and of strong build. Hypostatic patches were seen on the back side, and rigor mortis was present in all extremities. Apart from the single needle mark from the PVC, no injury was present on the body.

**Figure 3:**
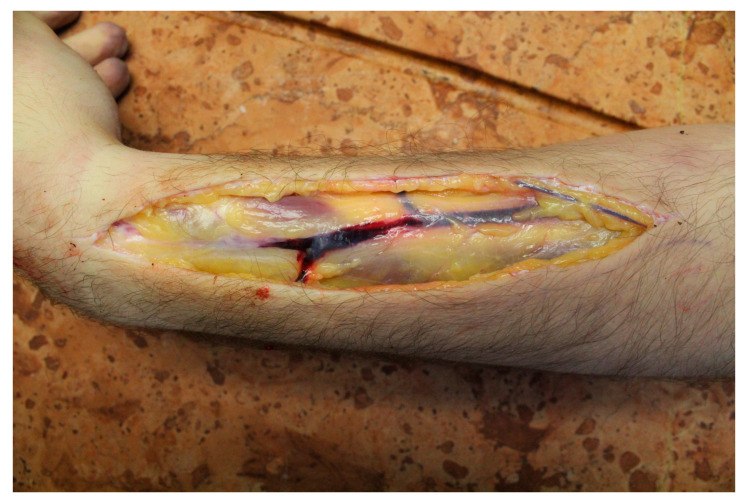
The subcutaneous tissues at the needle mark.

At the start of the internal examination, the superficial veins of the left forearm were examined, and one needle mark surrounded by minimal-sized fresh bleeding was observed (Figure 3). During the autopsy, brain edema (brain weight: 1410 g), general congestion of internal organs, severe dilation of heart chambers (heart weight: 339 g), and hypovolemic spleen were seen. The blood in the heart was mostly coagulated, while elsewhere, it remained mostly fluid. No sign of disease or injury was found. No macroscopic signs of putrefaction were seen. The histological examination showed severe lung congestion and edema, congestion of the liver and kidney, and mild steatosis in the liver.

### 2.2. Toxicological and Biochemical Analysis

The following samples were secured for toxicological analysis: blood from the femoral vein (FV), blood from the peripheral vein at the injection site (IS), blood from the left ventricle (LV) of the heart, urine, cerebrospinal fluid (CSF), vitreous humor (VH), and fluid from the drop chamber (DC) of the infusion set.

Biological samples were prepared by subzero-temperature liquid, with liquid extraction prior to analysis. Toxicological analysis was performed by two independent methods. TOX.I.S. II. System (Shimadzu HPLC module with UV spectral library) was used for the sample screening. Confirmatory test and quantitation were carried out using the SFC-MS/MS technique (Waters Acquity UPC2 supercritical fluid chromatograph with Waters Xevo TQ-S triple quadrupole mass detector). Alcohol concentration was determined from blood and urine samples using HS-GC-FID (Agilent Technologies 7890A headspace gas chromatography with flame ionization detector).

Biochemical analysis was performed using COBAS INTEGRA^®^ 400 plus (ion-selective electrode, automatic dilutated sample; Calibrator: ISE Reference Solution 1, 2 (Roche); Control: PreciControl ClinChem Multi1, 2 (Roche)).

The toxicological analysis of the blood from the femoral vein and the urine identified paracetamol (11 ng/mL blood and 84 ng/mL urine), theophylline (250 ng/mL blood and 1408 ng/mL urine), caffeine (994 ng/mL blood and 1224 ng/mL urine), and tramadol (146 ng/mL blood and 971 ng/mL urine). Ethyl-alcohol was not present in the samples. The results of post-mortem biochemical analyses are shown in Table 1.

## 3. Review of Literature

To estimate the threshold for a lethal dose of potassium, the cases reported in the scientific literature were reviewed. The literature search criteria were suicides using potassium (all successful and unsuccessful suicides) and hyperkalemia due to potassium overdose with high doses or antemortem (AM) concentrations. Suicidal overdose cases (irrespective of dose or blood concentration) and non-suicidal overdose cases with AM potassium concentration reaching at least 8 mEq/L were reviewed. Then, 8 mEq/L was chosen as a threshold for selection because the literature suggests hyperkalemia has fatal effects above this level [2,12]. Table 2 contains all potassium overdose cases with a dose and/or concentration above 8 mEq/L reported [5,6,8,9,11,16,17,18,19,20,21,22,23,24,25]. The lowest dose was 14.68 mEq for the reported lethal intravenous potassium overdose [16] and 283 mEq for the reported lethal oral overdose.

Only a few cases of suicide by potassium overdose are reported in the literature [6,7,8,9,10,11,19,22,23,25,27]. These cases were reviewed and summarized in Table 3 (cases were excluded if it was not clear whether the overdose was a result of suicide or an accident).

Of the five cases of attempted intravenous (IV) suicide, four cases succeeded [6,7,10]. The only victim who survived the suicide attempt was successfully resuscitated after being in cardiac arrest for five minutes [14]. In cases of suicide attempts by oral potassium, the victims were using their own potassium medications. Höjer et al. report a case of a woman with three separate unsuccessful episodes of suicidal potassium capsule overdose [22].

Only a few reports of potassium overdose cases describe macroscopic autopsy findings. Watanabe reported a double-suicide case with potassium infusion, where an autopsy of the male revealed lung congestion and edema, congested organs, 500 mL blood in the heart, and remarkable coagulation of the blood in the aorta pulmonary artery and vena cava inferior. The female victim showed lung edema, congested organs, fluid blood at every location, and a finger-tip-sized hemorrhage in the septum of the heart [6]. Bhaktanade reported a case of potassium overdose with lung edema and liver, spleen, kidney, and brain congestion [7].

Coulibaly et al. studied the macro-and microscopic changes in fetuses after the medical termination of pregnancies with KCl. In fetuses that received KCl injection, they noticed salt deposits in internal organs on macroscopic examination, along with crystals in several tissues: endocardium, epicardium, myocardium, liver, adrenal gland, testicle, spleen, kidney, and lung [28]. However, no other author reported similar findings, which can be explained by the lower potassium dose and concentrations used in lethal overdose cases in adults.

One feasible way to prove potassium overdose is to verify severe hyperkalemia. This can be undertaken easily in living victims but is a much harder task to do post-mortem. The post-mortem potassium level in different body fluids in lethal potassium overdose cases is reported in the literature in Table 4. Most authors use vitreous humor (VH) analysis, and—apart from the case we are presenting—only one author [2] measured the post-mortem potassium concentration in blood and vitreous fluid simultaneously.

The correlation between the potassium concentration of vitreous fluid and post-mortem interval (PMI) is well known, and there are several different methods for calculating the PMI from vitreous potassium concentrations [29,30,31,32,33,34,35,36,37,38,39,40]. This correlation can be used not only for estimating the time of death, but by reversing the calculations, these can also be used to predict the “normal” potassium level belonging to that PMI if the time of death is known [16]. Theoretically, if the measured vitreous fluid potassium concentration is well above the predicted normal value for that PMI, it can be a strong indicator of possible potassium intoxication. Table 5 describes the comparison of post-mortem vitreous fluid potassium levels in fatal overdose cases reported in the literature and the normal potassium level predicted by PMI. The calculations were made by methods with the fastest and slowest rise of post-mortem vitreous potassium concentrations reported by the authors [31,38]:

Sturner et al. [30]: PMI (hours) = (7.14 × K^+^ (mEq/L)) − 39.1

Jashani et al. [37]: PMI (hours) = 1.076 (K^+^ (mEq/L)) − 2.81

## 4. Discussion

Most cases of potassium overdose occur accidentally in hospital settings due to high-concentration potassium injections. Eliminating prescription use and use of undiluted KCl, use of prediluted formulations of 100–400 mEq/L and strict storage and handling rules for KCl ampules, along with rigid guidelines, can help to reduce the risk of possible accidental overdoses [1,5]. Poorly designed packaging can also lead to accidental potassium overdose, which can be avoided by standardizing the units in packaging and prescriptions [41]. Most suicidal potassium overdose cases are carried out with potassium chloride tablets [8,11,12,19,22,23,27], which are available in some countries without prescription and can be easily obtained online [27]. The rate of success for this suicide method is low. Most patients survive because the ingestion of slow-release potassium tablets increases the blood potassium level slowly, providing more time for therapeutic interventions, such as gastric evacuation [22], the administration of calcium gluconate, glucose, and insulin, potassium exchange resins, hemodialysis, and cardiac pacing [12,42,43]. The gradual increase in serum potassium levels pushes the threshold of lethal serum potassium concentration to a higher level than in intravenous use [26].

The highest survived serum potassium level was caused by intravenous administration of the wrong medication in a hospital setting in an infant who needed resuscitation but survived the incident without neurological deficit [18]. Survivors of hyperkalemia have been reported with serum potassium concentrations reaching 14 mEq/L [44] and 11.8 mEq/L [45]. Survivors of severe cases of hyperkalemia caused by renal failure have been reported as having serum potassium levels reaching 9.7 mEq/L and 10.2 mEq/L [46,47].

Suicide by intravenous administration of potassium is rare, but the chance of survival is low in these cases. The possibility of survival materializes in these cases only when professional help arrives in a brief time [14]. This type of suicide requires medical knowledge, experience, and access to medical supplies and so is carried out exclusively by medical professionals (Table 3).

Diagnosing a lethal potassium overdose presents a challenging task for the forensic pathologist. First, all other potential causes of death must be excluded, and then the potassium overdose itself must be somehow proved. Macro- and microscopic autopsy findings in potassium overdose cases are nonspecific: congestion of internal organs and lung edema are the only findings that are common in all cases. The specific finding of KCl deposits reported by Coulibaly in fetuses [28] is not reported by any other author. This leaves the diagnosis up to biochemical examinations. In a living person, 98% of body potassium is located in the cells [12], so the intracellular level of potassium is high (~150 mEg/L) [30] while the extracellular potassium level is much lower (serum: 3.8 to 5.5 mEq/L) [5]. The active membrane transport ceases, and the selective membrane permeability is disrupted due to the breakdown of cell membranes after death [30,48]; thereby, the potassium from the cells rapidly enters the extracellular fluid. Therefore, the potassium concentration will rise rapidly, largely in those tissues, which contain a high number of cellular elements (such as blood). The post-mortem serum potassium concentration does not reliably indicate the terminal serum-potassium level, even if PMI is only five minutes [49]. The post-mortem increase rate of serum potassium is not predictable, so the post-mortem blood potassium level is not indicative of the antemortem serum potassium level [49,50]. The post-mortem potassium concentration is between 25 and 84 mEq/L [10], and if we compare it with the fact that an increase of about 5 mEq/L (from 5 to 10 mEq/L) of blood potassium concentration is lethal, it becomes obvious that post-mortem blood potassium analysis cannot prove a potassium overdose. The presented case complements this statement with the uselessness of comparing the peripheral or cardiac potassium level in blood and the potassium level in the blood from the injection site.

After death, the potassium diffuses from the brain to the cerebrospinal fluid (CSF), increasing the potassium level of CSF [51]. Researchers state [52,53] that the rise is linear enough to use for the determination of the time of death, but one notes that it is reliable only in the first 20 post-mortem hours [50]. There is limited data about CSF potassium concentration in overdose cases: Palmiere reported much higher (~10 times) concentration in CSF than in vitreous fluid or serum [2], which indicates the low value of CSF potassium levels in overdose cases.

As our summary of post-mortem vitreous fluid concentrations reported in the literature shows, the potassium concentration of vitreous fluid cannot be used to prove potassium overdose: in many cases, it is below the predicted concentrations of deceased individuals without potassium overdose with the same PMI (Table 5), and there is even a case [2] in which the post-mortem potassium concentration does not even exceed normal potassium levels. The results can be improved if the potassium levels are also calculated for other factors, such as age or temperature [28], but there are cases indicating that great caution is always needed when assessing post-mortem vitreous fluid potassium levels [2]. The variations in pre-analytical treatment and in analytical methods (different techniques and instrumentation) can greatly affect the results of post-mortem vitreous fluid analysis [29,54]. Different ophthalmological pathologies or histories of previous surgical manipulation can also alter the vitreous fluid biochemistry. Therefore, only vitreous fluid biochemistry from “healthy” eyes should be analyzed [55]. These factors raise questions about the reliability of post-mortem vitreous fluid biochemistry since the removal of the eye only for excluding ophthalmological pathologies is usually out of the question, and previous ophthalmological history is not always known in cases of forensic autopsies. Other factors, such as ambient temperature, alcohol concentration, and renal failure reflected by urea nitrogen retention, also affect post-mortem vitreous potassium levels [29,50]. It is important to point out that in cases when the victim dies very rapidly from cardiac arrest due to injection of a large dose of potassium, there is not always enough time for the balance between the serum and vitreous fluid potassium levels to be reached [27,56]. The discrepancies in different studies regarding post-mortem change of vitreous fluid potassium concentration also have to be considered: most studies show a linear rise in potassium concentration [28,29], but there is a study also available stating that the rise is not entirely linear [57], and one that found no significant correlation between vitreous potassium level and PMI [58]. Overall, the reliability of post-mortem measurement of vitreous fluid potassium concentration is questionable, and it should be used in a forensic setting only if its limitations are known and revealed by the forensic pathologist [59].

Due to the factors discussed above, the most important method for establishing a diagnosis of potassium overdose is via circumstantial evidence. The presence of potassium tablets in the stomach, empty/partially empty boxes of potassium tablets at the scene, and the presence of an infusion set or syringe are important signifiers. In intravenous overdose cases, it is especially important to analyze the remaining fluid in the syringe, infusion bottle, drop chamber, or infusion tube [6,16].

In the presented case, all other potential causes of death were excluded: no disease was found, the medications did not reach toxic blood concentration [60], and the high potassium concentration from the drop chamber of the infusion set proved that the cause of death was potassium overdose.

## 5. Conclusions

The threshold of lethal potassium dose is around 15 mEq for intravenous and 300 mEq for oral overdose, but in cases of oral overdose, even much larger doses reaching 1000 mEq can be survived if treated in time. The threshold for lethal potassium blood concentration is 9–10 mEq/L.

Because of the factors described above, it can be stated that the potassium level at the time of death cannot be assessed reliably by the post-mortem potassium level of different body fluids [2,61,62], so post-mortem biochemical analysis has only limited diagnostic value in potassium overdose cases. The best feasible way to prove potassium overdose is the collection and analysis of circumstantial evidence, which underline the importance of accurate death scene investigation.

## Figures and Tables

**Table 1:** Results of biochemical analysis.

Sample	Na+ (mEq/L)	K+ (mEq/L)	Cl− (mEq/L)	Mg+ (mEq/L)	Ca+ (mEq/L)
FV	<50	62	<50	0.08	0.13
IS	57	75	73	0.35	0.08
LV	<40	45	67	0.10	0.00
urine	145	72	132	0.25	0.00
CSF	122	71	103	0.00	0.00
VH	195	22	135	0.33	0.23
DC	-	1250	-	-	-

FV: femoral vein, IS: injection site, LV: left ventricle, CSF: cerebrospinal fluid, VH: vitreous humor, DC: drop chamber.

**Table 2:** Potassium overdose cases in the literature with doses and/or ante-mortem K+ concentration ^1^ reported.

Author (Case No)	K+ Dose (mEq/L)	RoA	AM K+ cc (mEq/L)	Survived (Yes/No)
Watanabe (a) [6]	55	IV	N/A	No
Watanabe (b) [6]		IV	N/A	No
Wetherton (a) [5]	40	IV	N/A	No
Wetherton (b) [5]	26.4	IV	N/A	No
Wetherton (c) [5]	N/A	IV	12.4	No
Chaturwedi [16]	14.68	IV	N/A	No
Restuccio [8]	283	oral	9.6	No
Saxena [9]	800	oral SR	9.6	No
Kaplan [11]	540–720	oral	N/A	No
Wetli (a) [17]	N/A	oral SR	10.1	No
Wetli (b) [17]	N/A	N/A	10.8	No
Horisberger [18]	N/A	N/A	17.7	Yes
Su (a) [19]	1000	oral SR	9.7	Yes
Illingworth (a) [20]	168	oral SR	9.1	Yes
Illingworth (b) [20]	320	oral SR	9.3	Yes
Illingworth (c) [20]		oral SR	8.9	Yes
Steedman [21]	480	oral SR	9.1	Yes
Höjer [22]	1000	oral SR	9.2	Yes
Bosse [23]		oral SR	11	Yes
Nilsson [24]	300	oral SR	10.3	Yes
Iijama [25]	240	oral SR	7.0	Yes
Madan (a) [26]	239	oral ^1^	9.4	Yes
Madan (b) [26]	1237	oral ^1^	9	Yes
Madan (c) [26]	798	oral ^1^	8.4	Yes
Madan (d) [26]	1601	oral ^1^	8.2	Yes

^1^ not specified, RoA: Route of administration, IV: intravenous, SR: slow-release, AM: ante-mortem, N/A: no data available.

**Table 3:** Suicide by potassium overdose cases reported in the literature.

Author (Case No)	Sex (M/F)	Age (year)	Body Weight (kg)	Dose (mEq/L)	ROA	K+ cc (mEq/L)	Survived (Yes/No)	Profession
Watanabe (a) [6]	M	N/A	72.5	55	infusion	N/A	No	N/A
Watanabe (b) [6]	F	N/A	45.1		infusion	N/A	No	N/A
Bhatkande [7]	F	26	N/A	N/A	infusion	N/A	No	nurse
Bertol [10]	M	41	N/A	N/A	injection	N/A	No	nurse
Battlefort [14]	F	20	N/A	N/A	injection	3.9	Yes	nurse student
Restuccio [8]	M	53	N/A	283	oral SR	9.6	No	N/A
Saxena [9]	F	46	50	800	oral SR	9.6	No	N/A
Kaplan [11]	M	84	N/A	540	oral SR	N/A	No	N/A
Schaeffer [27]	M	30	93	400	oral SR	7.5	Yes	pharmacy ass. ^1^
Bosse [23]	F	56	N/A	N/A	oral SR	11	Yes	N/A
Su (a) [19]	F	50	N/A	1000	oral SR	9.7	Yes	N/A
Su (b) [19]	M	17	N/A	200–300	oral SR	6.1	Yes	N/A
Höjer [22]	F	28	N/A	1000	oral SR	9.2	Yes	N/A
Iijama [25]	F	26	N/A	240	oral SR	7.0	Yes	N/A

^1^ pharmacy assistant, RoA: route of administration, SR: slow-release, N/A: no data available.

**Table 4:** Potassium concentration in post-mortem body fluid samples and in the circumstantial evidence.

Author (Case No)	PMI (hour)	LV (mEq/L)	FV (mEq/L)	CSF (mEq/L)	VH (mEq/L)	Urine (mEq/L)	Evidence (mEq/L)
Watanabe (a) [6]	34	49.7	N/A	N/A	N/A	N/A	690–746
Watanabe (b) [6]	34	62.8	N/A	N/A	N/A	N/A	
Wetherton (a) [5]	N/A	N/A	N/A	N/A	1.9 ^1^	N/A	N/A
Wetherton (b) [5]	5.5	N/A	N/A	N/A	9.5	N/A	N/A
Wetherton (c) [5]	13.5	N/A	N/A	N/A	9.3	N/A	N/A
Chaturvedi [16]	~48	N/A	N/A	N/A	8.9–9.5	21.8	1468
Bertol [10]	N/A	160	87	N/A	N/A	N/A	N/A
Palmiere [2]	4:45	7.1	6.9	43.4	4.3–5.7	57	N/A
Bhatkande [7]	N/A	77.753 ^2^		N/A	N/A	N/A	N/A
Presented case	70	45	62	71	22	72	1250

^1^ Analysed after 4 days of formalin fixation, ^2^ no source of blood samples reported, PMI: post mortem interval, LV: left-ventricle (cardiac blood), FV: femoral vein, CSF: crebrospinal fluid, VH: vitreous humor, NA: no data available.

**Table 5:** PMI and post-mortem vitreous fluid concentrations and predicted normal vitreous humor concentrations.

Author (Case No)	PMI (hour)	VH K+ cc (mEq/L)	Predicted Normal VH K+ cc by Sturner’s Calculation [31] (mEq/L)	Predicted Normal VH K+ cc by Jashani’a Calculation [38] (mEq/L)
Wetherton (b) [5]	5.5	9.5	6.24	7.72
Wetherton (c) [5]	13.5	9.3	7.36	15.157
Chaturvedi [16]	~48	8.9–9.5	12.19	47.22
Palmiere [2]	4:45	4.3–5.7	6.14	7.26
Wetli [17]	18	10.8	7.99	19.34
Presented case	70	22.5	15.458	72.66

PMI: post mortem interval, VH: vitreous humor.

## Data Availability

All data are contained within the article.
